# Principles for gene drive research: Sponsors and supporters of gene drive research respond to a National Academies report

**DOI:** 10.1126/science.aap9026

**Published:** 2017-12-01

**Authors:** Claudia Emerson, Stephanie James, Katherine Littler, Filippo (Fil) Randazzo

**Affiliations:** 1 Institute on Ethics and Policy for Innovation, McMaster University, Hamilton, Ontario L8S 4L8, Canada; 2 Foundation for the National Institutes of Health, North Bethesda, MD 20852, USA; 3 Wellcome Trust, London NW1 2BE, UK; 4 Bill & Melinda Gates Foundation, Washington, DC 20005, USA

The recent outbreak of Zika virus in the Americas renewed attention on the importance of vector-control strategies to fight the many vector-borne diseases that continue to inflict suffering around the world. In 2015, there were ~212 million infections and a death every minute from malaria alone (*1*). Gene drive technology is being explored as a potentially durable and cost-effective strategy for controlling the transmission of deadly and debilitating vector-borne diseases that affect millions of people worldwide, such as Zika virus and malaria. Additionally, its suitability is being evaluated for various potential applications in conservation biology, including a highly specific and humane method for eliminating invasive species from sensitive ecosystems (*2*, *3*). The use of gene drives is an emerging technology that promotes the preferential inheritance of a gene of interest, thereby increasing its prevalence in a population. A gene drive is distinct from genome editing, in which the genetic change is not preferentially inherited. A variety of gene drives occur in nature that can cause genetic elements to spread throughout populations to varying degrees, and researchers have been studying how to harness these to solve some of society’s most intractable problems (*4*). Aided by CRISPR gene-editing technology, the rapid pace with which the research is progressing is demonstrated by recent successes in laboratory experiments (*5*, *6*), although observation of resistance developing in one instance highlights the need for further research (*7*).

In recognition of the rapid advances of research in this field, the U.S. National Institutes of Health (NIH) and the Foundation for the NIH requested that the U.S. National Academies of Sciences, Engineering, and Medicine (NASEM) conduct a study that would “summarize current understanding of the scientific discoveries related to gene drives and their accompanying ethical, legal, and social implications,” which was published in 2016 [(*2*), p. vii)]. The authors noted that the promise of gene drives is tempered by uncertainties regarding potential for harm from unintended consequences or misuse of the technology. The potential persistence of genetic change in the target population caused by a gene drive is both the source of optimism for a durable and affordable tool to combat a variety of pernicious public health and environmental problems as well as the source of concern about the possibility for irreversible harm to the ecosystem that has prompted some to call for a moratorium on the research (*2*, *8*, *9*). This led the authors of the National Academies report to advocate for a precautionary contextual approach to the science— i.e., concluding that currently there is insufficient evidence to support deployment of gene drive–modified organisms into the environment but that the potential benefits justify proceeding with laboratory research and highly controlled field trials (*2*, *10*).

**Figure:**
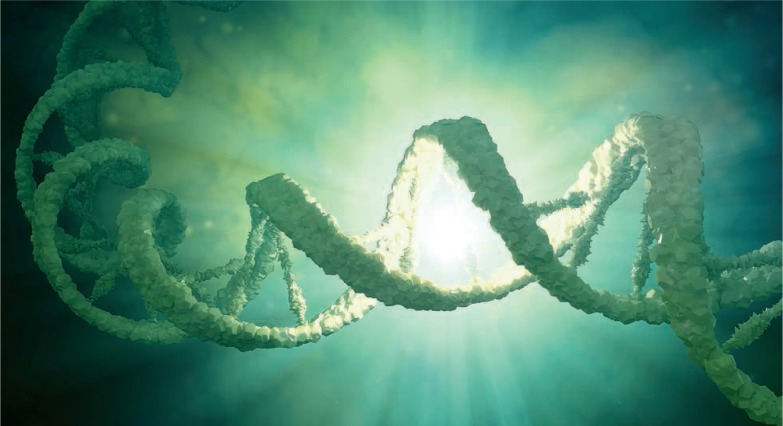


The report issues a number of recommendations aimed at researchers, funders, and policy-makers on actions important for minimizing potential risks, averting preventable harm, and earning the confidence and support of the public. Of the 32 recommendations made, 13 are specific to funders—including one aimed specifically at “United States funders” (*2*).

## RESPONDING TO THE NASEM REPORT

Sponsors of scientific research have a responsibility to support innovation that promotes and sustains the public good (*11*). They share the common goal of advancing knowledge and human well-being, while protecting and promoting societal values that underpin the responsible conduct of science. The 2010 report from The Presidential Commission for the Study of Bioethical Issues, “New Directions: The Ethics of Synthetic Biology and Emerging Technologies,” highlights the important point that the responsibility for ensuring the conduct of quality science is not the exclusive domain of scientists, but is a shared responsibility among research sponsors and policy-makers alike (*11*). In this Policy Forum, we use the term “science” in its broadest sense, referring inclusively to the life and physical sciences as well as social science, and the humanities, i.e. ethics. Moreover, researchers, sponsors, and policy-makers also share the responsibility of monitoring the progress of science and communicating it effectively to the public (*2*). Effective public engagement, underpinned by transparent dialogue around both the potential benefits and risks, is critical for enabling well-informed public discussion and debate that is free from the type of sensational hype that has framed new technology in the past (*12*).

As sponsors and supporters of gene drive research, the signatories to these principles have come together to provide a coordinated response to the NASEM recommendations in the form of commitment to a set of guiding principles (see the box) intended to (i) mobilize and facilitate progress in gene drive research by supporting efforts of the highest scientific and ethical quality; (ii) inspire a transparent atmosphere of conscientiousness, respectfulness, and integrity wherein the research can flourish; and (iii) support existing biosafety requirements and best practices as minimum standards for research. Endorsement of the principles represents a pledge to advance the foundational elements of efficient and responsible research conduct: evidence, ethics, and engagement, which are also important themes represented throughout the NASEM report.

The principles are presented in the box, with references indicating the NASEM recommendation to which the principle responds.

## AN ETHIC OF RESPONSIBILITY

Through alignment with the principles, sponsors of gene drive research aim to contribute to an adaptive and data-informed toolbox of policies that can support the responsible development of gene drive research [(*2*), p. 172]. Such a toolbox affords the flexibility to respond to new technical advances and knowledge, while ensuring the long-term safety of human health and the environment. Principles serve as a moral compass to “anchor the actionables,” so that only the highest-quality research endeavors, consistent with the best-practice guidance and standards set by the scientific community, will be supported. As the NASEM report notes, “institutions, funders, and professional societies work in concert to encourage professional best practices in research. Such cooperation will be instrumental to maintaining high standards in gene drive research” [(*2*), p. 8].

To date, 13 organizations have endorsed the principles, and other sponsors and research organizations in both the public and private sector are encouraged to contact the corresponding author if they wish to sign on. The signatories to the principles will cooperate on catalyzing a culture of responsible innovation by encouraging sponsors in the public and private sectors to endorse and implement the guiding principles in funding decisions and research management. Moving forward, the forum of gene drive sponsors and supporters will convene to discuss next steps in operationalizing the principles. Although there are many challenges to address, the forum will start with consideration of harmonized approaches to stakeholder engagement, regulatory oversight, transparency and data sharing to support the research, knowledge sharing, and public discourse on gene drive technology. The forum is in a position to develop a “consensus standard” designed to set an agreed level of good practice or quality to help establish confidence in gene drive innovations, and to continue working with stakeholders and relevant agencies to implement all of the principles. This will ensure progress, efficiency, and a common framework within which to move the field forward.

**Guiding principles for the sponsors and supporters of gene drive research**

**Advance quality science to promote the public good**

The pursuit of gene drive research must be motivated by, and aim to promote, the public good and social value. Funded research shall embody the highest quality science and ethical integrity, consistent with the current best practice guidance set by the research community and relevant decision-making bodies [(*2*), p. 106)].

**Promote stewardship, safety, and good governance**

Researchers and sponsors are stewards of science and the public trust. It is imperative that good governance is demonstrably shown in all phases of the research, and especially in relation to risk assessment and management. This requires compliance with applicable national and international biosafety and regulatory policies and standards. Research conducted with respect and humility for the broader ecosystem in which humans live, taking into account the potential immediate and longer-term effects through appropriate ecological risk assessment, is a hallmark of both good stewardship and good governance [(*2*), pp. 128; 170–172)].

**Demonstrate transparency and accountability**

Knowledge sharing is not only essential for the advancement of science, but for transparency to foster public trust in emergent technologies. The timely reporting of results and broad sharing of data shall be the norm in gene drive research, consistent with the tradition of openness established in its parent communities of genetic and genomic science. Measures of transparency and accountability that contribute to building public trust and a cohesive community of practice will be supported [(*2*), pp. 171; 177–178)].

**Engage thoughtfully with affected communities, stakeholders, and publics**

Meaningful engagement with communities, stakeholders, and publics is critical for ensuring the best quality science and building and sustaining public confidence in the research. Funded research shall include the resources needed to permit robust, inclusive, and culturally appropriate engagement to ensure that the perspectives of those most affected are taken into account [(*2*), pp. 142–143)].

**Foster opportunities to strengthen capacity and education**

Strengthening capacities in science, ethics, biosafety, and regulation is essential for enabling agile and steady progress in gene drive research globally. Opportunities to partner, educate, and train shall be supported throughout all phases of the research, from the early stages to deployment. Strengthening capabilities within countries for testing and deploying the technology is essential for informed decision-making [(*2*), pp. 128; 170–172)].
